# Ozone: Unrealistic Scenarios

**DOI:** 10.1289/ehp.113-1277873

**Published:** 2005-02

**Authors:** Joel Schwartz, Patrick Michaels, Robert E. Davis

**Affiliations:** American Enterprise Institute for Public Policy Research Washington, DC, E-mail: jschwartz@aei.org; Department of Environmental Sciences University of Virginia Charlottesville, Virginia

Knowlton et al. (2004) argued that increasing temperatures associated with climate change will increase urban ozone and related health risks. They have disregarded important factors in reaching this conclusion.

During the last 20 years, nationwide exceedances of the federal 1-hr ozone standard declined 90%, and the June–August average of daily 1-hr peak ozone levels declined 10% (Schwartz et al., in press), presumably with ensuing declines in ozone-related mortality. Ozone declined despite a roughly 1°C increase in urban temperatures during the last few decades (Karl et al. 1988). Knowlton et al. (2004) did not explain why we should expect the future to be the opposite of the past.

Knowlton et al. (2004) used ozone-precursor [nitrogen oxides (NO_x_) and volatile organic compound (VOC)] emissions estimates for 1996 to predict ozone levels in the 2050s. However, even current emissions are substantially lower than 1996 levels, while, as shown below, already-adopted U.S. Environmental Protection Agency (EPA) requirements will eliminate most remaining ozone-precursor emissions, even after accounting for growth.

The U.S. EPA (2003) estimated that between 1996 and 2001, total emissions of NO_x_ and VOC declined, 10 and 14%, respectively. [The U.S. EPA updated its trend estimates in November 2004 (U.S. EPA 2004a) and now believes the decline was even steeper, although these new estimates were obviously not available to Knowlton et al.] During 2003 and 2004, the U.S. EPA capped total NO_x_ from coal-fired power plants and industrial boilers at 60% below 2000 levels (U.S. EPA 1998a, 2004b). A range of emissions data show the average automobile’s NO_x_ emissions rate declined 4–9% per year between 1995 and 2001, with greater improvements for vehicles up to 4 years old (Pokharel et al. 2003; Schwartz 2003). Total driving is increasing < 2% per year, resulting in large net NO_x_ declines (Texas Transportation Institute 2004).

Data on heavy-duty diesel vehicles are sparse, but there is every reason to believe that diesel NO_x_ has also declined. The U.S. EPA has tightened NO_x_ standards for new on- and off-road diesels several times over the last 15 years, and also recently required additional NO_x_ reductions from in-use 1993–1998 model year trucks (U.S. EPA 2002a, 2004c, 2004d).

VOCs have declined far more than NO_x_ and far more than U.S. EPA estimates. The U.S. EPA’s official VOC inventory understates significantly the gasoline-vehicle contribution to total VOCs (Watson et al. 2001). Real-world data show the average automobile’s VOC emission rate is declining 11–15% per year, again much more rapidly than driving is increasing, and with a more rapid decline for recent models (Pokharel et al. 2003; Schwartz 2003). The U.S. EPA also recently implemented VOC reductions for other sources (U.S. EPA 1998b, 2002b, 2004e).

Overall, between 1996 and 2004, anthropogenic NO_x_ and VOC emissions likely declined, respectively, at least 25 and 50%—declines overlooked by Knowlton et al. (2004).

Emission declines will continue. For example, a vehicle fleet meeting the U.S. EPA’s “Tier 2” automobile standards, implemented in 2004, on-road diesel standards set for 2007, and off-road diesel standards set for 2010, will emit 90% less NO_x_ and VOCs per mile over their lifetime than the current average vehicle, resulting in huge emissions declines, even with predicted increases in driving (Schwartz 2003; U.S. EPA 2000a, 2000b, 2004c).

Knowlton et al. (2004) assume ozone-precursor emissions several times greater than any plausible future scenario. Their projections of future ozone and related health impacts are therefore unrealistically high.

Heat-related mortality has also declined, by 70% nationwide since the 1960s, despite warming urban climates, with the hottest and most humid cities achieving the greatest risk reductions (Davis et al. 2003). These health improvements resulted from a range of adaptive technologies and processes, including increased air conditioning, changes in building design, physiologic adaptations, and improved emergency medicine.

Nevertheless, because of a single major blackout on a warm day in 2003, Knowlton et al. (2004) maintain that “air conditioning may not really be an appropriate ‘fix’ for adapting to climate change.” Air conditioning is clearly a vital adaptive technology that has saved countless lives. One study reported a relative risk of death on hot days of 1.7 for people with no air conditioning compared to those with central air (Rogot et al. 1992).

The nondiscriminating reader might be impressed by the downscaling of a general circulation model using a regional mesoscale model to predict localized differences in future air-pollution related mortality, but the complexity of the models is irrelevant in the face of Knowlton et al.’s failure to temper their theoretical exercise with real-world data. Had Knowlton et al. (2004) accounted for observed historical health and pollution trends and future emission-reduction requirements, they would have arrived at a markedly different story.
